# Complete mitochondrial genome analyzes of four gerbil species (Rodentia: Gerbillinae) distributed in Türkiye

**DOI:** 10.7717/peerj.21330

**Published:** 2026-06-16

**Authors:** Derya Çetintürk

**Affiliations:** Department of Biology, Faculty of Science, Ankara University, Ankara, Turkey

**Keywords:** Meriones, Gerbil, Mitogenome, Türkiye

## Abstract

**Background:**

The genus *Meriones* Illiger, 1811 is widely distributed in the Middle East, Northern Africa, and Central Asia. In spite of many studies evaluating the gerbil phylogeny, the systematics of this genus is controversial because studies analyzing a single gene region yield inconsistent results. The presented research provides the first complete mitogenomes of four gerbil species (*Meriones crassus, Meriones persicus, Meriones tristrami,* and *Meriones vinogradovi*) distributed in Türkiye and novel and strong results to clarify the phylogenetic relationships of these gerbils.

**Methods:**

Next Generation Sequencing (NGS) was performed using the specimens from Türkiye. Other gerbil species (*M. tamariscinus, M. meridianus (M. dahli), M. unguiculatus, M. libycus, Rhombomys opimus, Psammomys obesus, Brachiones przewalskii,* and *Gerbilliscus leucogaster*) were also included in the phylogenetic analyses. In addition to determining the mitogenome characteristics, mean genetic distance values, haplotype and nucleotide diversity values, numbers of mutations and polymorphic sites were calculated, and a Bayesian Inference and Maximum Likelihood trees were constructed along with the divergence times.

**Results:**

Most of the 37 gene regions were encoded on the H-strand; the GC% ratio varied between 36.9 and 38.1% among the four species that show their genomes were AT-rich. Bayesian Inference and Maximum Likelihood trees gave well-supported genetic relationships: *P. obesus* and *G. leucogaster* were located as the most distant species, and *M. tamariscinus* and *R. opimus* were found to be closer. *M. meridianus, M. unguiculatus,* and *M. persicus* and *M. tristrami*, *M. crassus*, *M. libycus*, and *M. vinogradovi* formed two differentiated lineages. In the latter lineage, *M. tristrami* and *M. crassus* were clustered. Also, *B. przewalskii* was linked to the former two lineages. In addition, mean genetic distance values between *Meriones* species were determined to be 7.04–16.59%, in accordance with the results of the phylogenetic approach. It was also calculated that the species mentioned above diverged 11.04 MYA (95% HPD: 7.94–14.68), corresponding to the Late Miocene Epoch.

**Conclusion:**

In contrast to previous studies, this study supports the paraphyly of subgenus *Pallasiomys* regarding the *M. persicus*’ position with high reliability results. Besides, the paraphyletic status of the genus *Meriones* was proved with the phylogenetic relationship of *B. przewalskii* and *Meriones* species. It was also emphasized that mitogenome analyses are notably effective in order to study problematic systematics of rodent groups like *Meriones*.

## Introduction

*Meriones* Illiger, 1811 is a rodent genus represented by 17 species in desert and steppe habitats and distributed from North Africa to the Arabian Peninsula, from the Anatolian Peninsula to the Caucasus, Mongolia, and China (Musser & Carleton, 2005; Chevret & Dobigny, 2005; Naseri et al., 2006; Ito et al., 2010; Wilson, Lacher Jr & Mittermeier, 2017). Until now, five gerbil species have been recorded in Türkiye: *Meriones crassus* Sundevall, 1842; *Meriones persicus* Blanford, 1875; *Meriones tristrami* Thomas, 1892; *Meriones vinogradovi* Heptner, 1931, and *Meriones dahli* Shidlovsky, 1962 (Kefelioğlu, 1997; Yiğit, Kıvanç & Çolak, 1997; Yiğit, Kıvanç & Çolak, 1998; Yiğit & Çolak, 1999). *M. vinogradovi* and *M. tristrami* are quite similar to each other in terms of external morphology and skull characteristics. *M. crassus* differs from the other species in that the auditory capsules are larger, the posterior part of the suprameatal triangle is open, and the brain capsule is wide (Yiğit, Kıvanç & Çolak, 1997; Yiğit, Kıvanç & Çolak, 1998; Yiğit et al., 2006a). On the other hand, although *M. crassus* and *M. dahli (M. meridianus)* are similar in size, they differ in fur color, distribution areas, and habitat preferences.

Genus *Meriones* includes four subgenera as *Meriones* s.str., *Parameriones, Pallasiomys,* and *Cheliones* based on morphological features. Subgenus *Meriones* has only one species *M. tamariscinus* differ from other subgenera in very specific male genital morphology (Pavlinov, 1982; Pavlinov, 1986; Pavlinov et al., 1990). *Pallasiomys* species show the specific genital morphology (Pavlinov, 1986). *M. persicus* from *Parameriones* morphometrically separates from *Pallasiomys* species (*M. crassus, M. tristrami, M. vinogradovi,* and *M. dahli (M. meridianus)*) (Yiğit et al., 2018).

A significant part of the phylogenetic studies on this genus comprises allometric and morphological methods (Benazzou et al., 1984; Darvish, 2009; Yiğit et al., 2013; Yiğit et al., 2016; Yiğit et al., 2018; Dianat et al., 2017). In studies based on molecular markers, the results presented by different authors significantly contrast with each other, causing problems and confusion regarding the systematics of the genus. Chevret & Dobigny (2005) studied the systematic and evolution of Gerbillinae based on genetic markers, such as *Cyt-b* and *12S rRNA*. They suggested that the subgenera of the genus *Meriones* are not monophyletic since *Meriones rex* and *M. crassus*, which belong to different subgenera, appear to be sister species in the Maximum Likelihood Tree. Also, *M. crassus/M. libycus* and *M. meridianus/M. unguiculatus* were found to be sister taxa. These results were confirmed by Ito et al. (2010) by using *Cyt-b* and *COII* data. *Cyt-b* data given by Bouarakia et al. (2021) showed *M. tristrami/M. rex/M. crassus, M. libycus/M. vinogradovi,* and *M. meridianus/M. chengi/M. unguiculatus* groups. Alhajeri, Hunt & Steppan (2015) employed some mitochondrial and nuclear markers and determined *M. libycus/M. tristrami/M. crassus/M. rex* clade. In addition, *M. persicus* and *M. tamariscinus* are connected to the studied *Meriones* species; *Psammomys obesus*, *Psammomys vexillaris, Brachiones przewalskii,* and *Rhombomys opimus* diverged from *Meriones*. Afzali & López-Antoñanzas (2023) found two main lineages according to *Cyt-b* analyzes (strongly supported *M. libycus/M. tristrami/M. crassus* and less supported *M. persicus/M. meridianus/M. vinogradovi/R. opimus* lineages). As can be inferred, the evolutionary relationships among *Meriones* species analyzed in this genus are incongruent, particularly with respect to the taxonomic position of *M. vinogradovi*. Genetic studies involving new methods are essential for these morphologically difficult-to-distinguish species.

Studies analyzing the complete mitochondrial DNA (mtDNA) rather than using specific gene regions have become ascendant in recent years. The mammalian mtDNA genome, approximately 16 kb long, contains 37 gene regions (Anderson et al., 1981). Due to the lack of recombination and repair mechanisms, the absence of introns, and a rapid evolutionary rate, mtDNA is frequently used in genetic, molecular systematic, and phylogeny studies (Clary & Wolstenholme, 1985; Wolstenholme, 1992; Nabholz, Glémin & Galtier, 2009; Fernández-Silva, Enriquez & Montoya, 2003; Ballard & Whitlock, 2004; Aanen, Spelbrink & Beekman, 2014). Until now, complete mtDNA information of four gerbil species (*M. tamariscinus, M. meridianus (M. dahli), M. unguiculatus,* and *M. libycus*) have been reported (Li et al., 2016a; Li et al., 2016b; Luo & Liao, 2016a; Luo & Liao, 2016b; Brekke et al., 2023). The complete mtDNA sequences of *M. crassus, M. persicus, M. tristrami,* and *M. vinogradovi* species were provided herewith, along with the genetic distance and phylogenetic assessments. In this frame, the complete mtDNA structure was characterized for the first time for the mentioned four species, and it was aimed to clarify the complex phylogeny of the genus *Meriones* as well as contribute to the data for rodent systematics and taxonomy in a broader context.

## Materials & Methods

### Sampling

**Figure 1:**
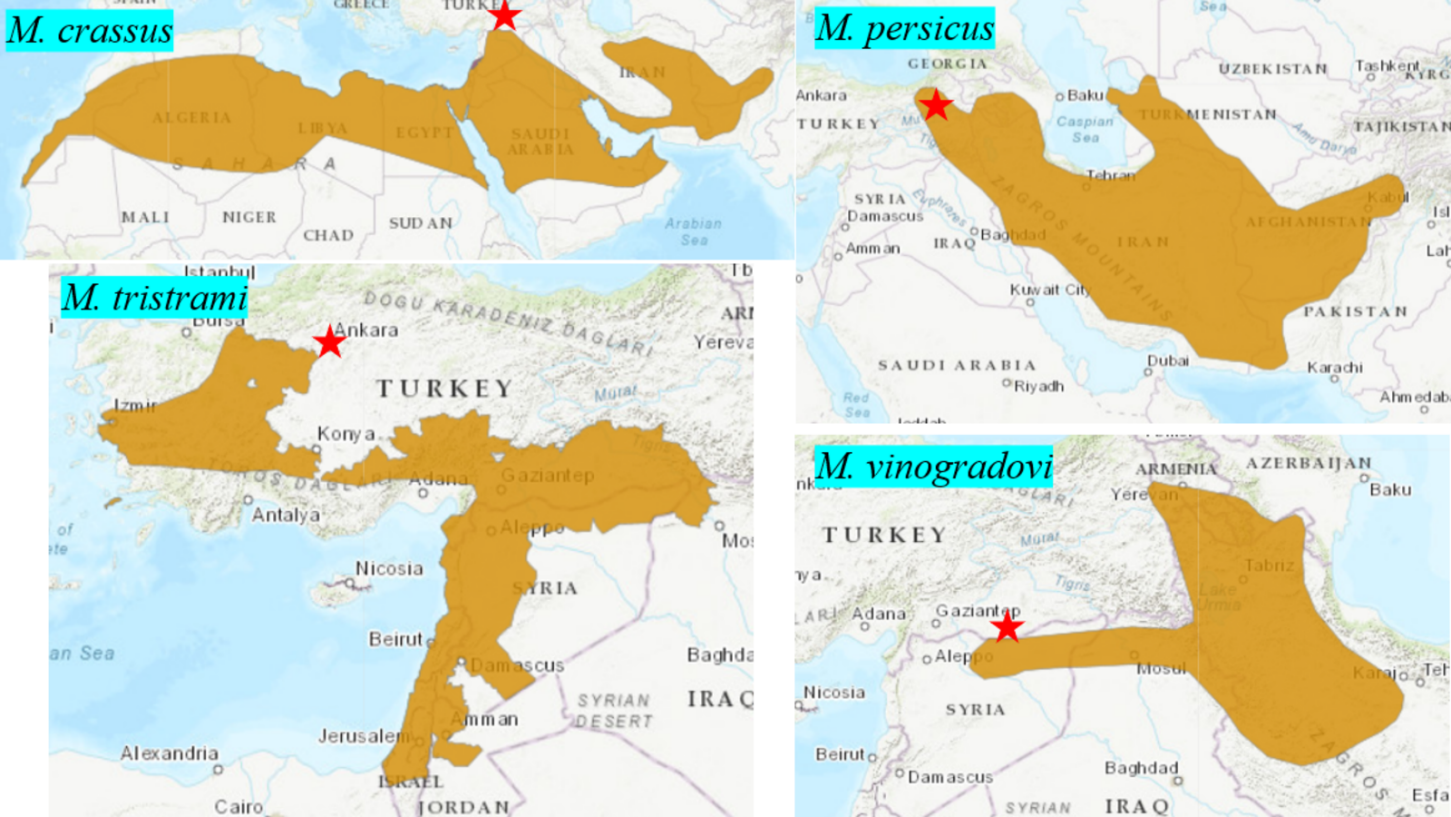
Sampling locations and distribution ranges of the studied *Meriones* species. Distribution maps adapted from IUCN (2024).

One specimen for each species, stored in the Ankara University Mammalian Research Collection (AUMAC, https://mammalia.ankara.edu.tr/), as tissues preserved in a deep freezer since the 1990s, was used for mtDNA analysis. *M. crassus* (Şanlıurfa Province−37°19′43″N, 39°01′35″E), *M. persicus* (Ağrı Province−39°43′07″N, 43°03′03″E), *M. tristrami* (Ankara Province−39°55′11.53″N, 32°51′15.37″E), and *M. vinogradovi* (Şanlıurfa Province−37°19′43″N, 39°01′35″E) samples (Fig. 1) were collected as dead specimens and identified according to their morphological characteristics (Yiğit et al., 2006a). Accordingly, *M. crassus* is distinguished from other species by its bulging auditory capsules in the skull, the open posterior part of the suprameatal triangle, and the wide brain capsule. *M. tristrami* and *M. vinogradovi* are very similar to each other especially in terms of external morphology, but the skull of *M. vinogradovi* is slightly more robust and longer and the averages of external and cranial measurements are notably longer than *M. tristrami*. *M. persicus* resembles *M. tristrami*; however, the white supraorbital spot in *M. persicus* is the main discrimitive characteristics.

Besides the samples mentioned earlier, *M. tamariscinus, M. meridianus (M. dahli), M. unguiculatus, M. libycus, Rhombomys opimus, Psammomys obesus, Brachiones przewalskii, Gerbilliscus leucogaster,* and as outgroups *Arvicanthis rufinus* and *Mus musculus* sequences were acquired from GenBank (https://www.ncbi.nlm.nih.gov/genbank/) (Table S1).

### Laboratory studies and genome analyzes

Genomic DNA was extracted from liver tissue using the QiAamp DNA Mini Kit (Cat. No: 51304; Qiagen), following optimized protocols designed explicitly for tissue-derived DNA isolation. The quality and concentration of the extracted DNA were assessed using the Qubit™ dsDNA Quantification Assay Kit (Cat. No: Q32851; Thermo Fisher Scientific) on the Qubit 4.0 fluorometer. Subsequent library preparation was executed employing the NEBNext Ultra DNA Library Prep Kit (Cat. No: E7370L; NEB), strategically optimizing paired-end sequencing with a precise 150-base pair insert size configuration. Sequencing was conducted utilizing the high-throughput Illumina NovaSeq 6000 platform.

The initial step in mitochondrial genome assembly and annotation involved assessing the quality of the reads obtained from the NGS run. Summary statistical data were generated to evaluate read quality and sequencing depth based on Sequence Quality Histograms and Per Sequence Quality Scores utilizing FastQC Version: 0.12.1 (Simon Andrews et al., 2010). Following this assessment, low-quality reads were filtered out, and a high-quality dataset in FASTQ format (“.fastq” or “.fq”) was prepared for downstream analysis. The fastq summary statistics provide essential insights into the quality and characteristics of the NGS reads generated from the eukaryotic whole-genome sequencing experiment. Following the sequence quality evaluation, the resulting FASTQ files were analyzed using FastQC and MultiQC tools. A three-step bioinformatics analysis was performed to elucidate the genetic composition, profile, and potential functionalities, including (i) quality control of NGS data and filtering, (ii) *de novo* genome assembly, (iii) mitochondrial genome annotations. Following the NGS sequencing reaction, the total number of reads, total sequence length, Phred Score (Q), Q20 and Q30 quality scores, and %GC contents were calculated. *Meriones* mitochondrial genome and contig sequences were obtained using a de novo genome assembly method using GetOrganelle v1.8.0.0 software (Jin et al., 2020, https://github.com/Kinggerm/GetOrganelle). Following successful de novo genome assembly, annotations were meticulously performed on the resulting sequences. Mitochondrial genome annotation was performed using MitoZ version 3.6. (Meng et al., 2019). The assembled circular genome and its annotations were visualized through MitoFish v2025.06 (Iwasaki et al., 2013; Sato et al., 2018; Zhu et al., 2023). Genoks LifeScience Inc. performed these stages in Türkiye. Haplotype diversity (Hd), nucleotide diversity (Pi), as well as the number of mutations and polymorphic sites were determined using DnaSP V6 (Rozas et al., 2017).

To execute phylogenetic analyzes, 16,469 base-pair complete mtDNA was obtained after the alignment process conducted in Mega11 Software (Tamura, Stecher & Kumar, 2021). Mean genetic distance (*d*) values between the sequences were calculated based on the *p*-distance (Hamming, 1950) parameter in this software. A saturation test was performed using DAMBE 7.2.1 Software (Xia, 2018), and little saturation was detected. Phylogenetic and divergence time analyses were conducted using the complete mitochondrial genome sequences of *Meriones* species, excluding the D-loop region. The D-loop region was removed prior to analysis due to its elevated substitution rate, frequent indels, and alignment ambiguity, which can bias branch length estimation and molecular clock inference (Brown, George & Wilson, 1986; Pesole et al., 1999; Nabholz, Glémin & Galtier, 2008). The best substitution models were identified (GTR+G for CDS and rRNA genes) with ModelFinder embedded in the IQTREE Web Server (https://iqtree.github.io/) to generate Maximum Likelihood (ML) and Bayesian Inference (BI) trees of complete mtDNA according to BIC (Bayesian Information Criterion). Establishing Bayesian tree and estimating the divergence time of gerbil species were conducted in the BEAST 1.7.5 Program (Drummond & Rambaut, 2007). As a calibration point, *Mus-Arvicanthis* split (mean = 11.2 Ma; SD = 0.3, Kimura et al., 2015) with a normal distribution were chosen. “Yule process of speciation” and “uncorrelated lognormal relaxed molecular clock model (UCLN)” models was accepted in the Beauti Program stored in BEAST 1.7.5 and the “xml” format file was prepared and analysed in five repetitions, each chain length being 20.000.000, which is sampled every 2,000 generations. The five formed files were combined with LogCombiner, and a tree was constructed in TreeAnnotator with a burn-in of 5.000. Stationarity and convergence were checked in Tracer 1.5 Software (http://beast.bio.ed.ac.uk/Tracer), and analysis results were taken into consideration only if all effective sample size (ESS) values were 200 or higher. Maximum Likelihood (ML) tree was constructed *via* the IQTREE Web Server (https://iqtree.github.io) with 1,000 ultrafast bootstrap. The (BI) and ML trees were visualized and modified in FigTree 1.4 (http://tree.bio.ed.ac.uk/software/figtree).

Similarly, *Cyt-b* analyses were also performed with sequences from GenBank (Table S1), and the BI and ML trees were constructed selecting the GTR+G (Tavaré, 1986) substitution model, and *d* values were calculated using the aforementioned method.

## Results

### Characterization of mitogenome structure of *Meriones* species

**Figure 2:**
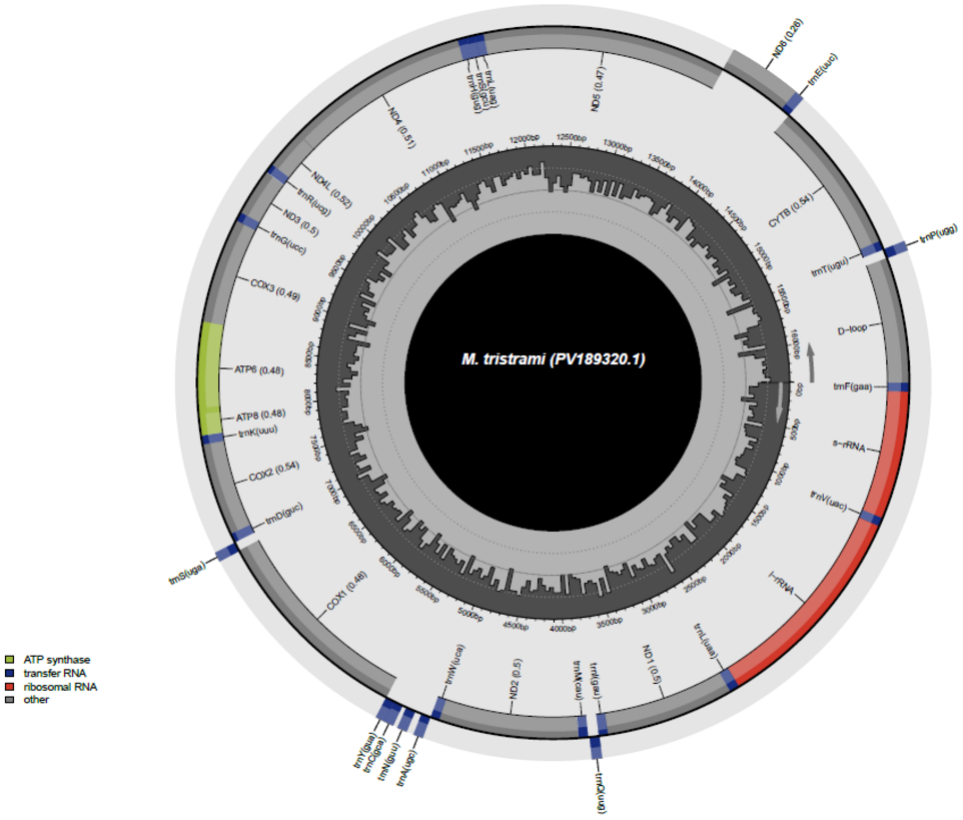
Mitogenome characteristics of *M. tristrami*.

Four previously morphologically identified gerbil species, whose mitogenomes were provided for the first time, were analyzed for the *Cyt-b* gene using NCBI-BLAST and confirmed to belong to the species, as shown in Table S2. For these species, 37 gene regions were successfully obtained, including 13 protein-coding genes, 22 tRNA genes, 2 rRNA genes, and 1 non-coding *D-loop* (*control*) region (Fig. 2, Figs. S1–S3). Considering the sequence lengths, 16,412, 16,368, 16,452, and 16,485 base-pair regions were obtained for *M. tristrami, M. persicus, M. vinogradovi,* and *M. crassus,* respectively. It can be seen that *tRNAPro, tRNAGlu, ND6, tRNASer, tRNATyr, tRNACys, tRNAAsn, tRNAAla,* and *tRNAGln* were located on the L strand, while the other regions were encoded on the H strand (Tables S3–S6). Length of protein-coding sequences (PCGs) ranged from 11,404 to 11,417 bp (69.20–69.76% of the complete data). Besides the length difference (1–6 nucleotides) observed in some regions (*e.g 12S rRNA, 16S rRNA, tRNA-Asn, ATP synthase F0 subunit 8 (ATP8), tRNA-Arg, NADH dehydrogenase subunit 6 (ND6), tRNA-Pro,* and *Cyt-* b), some genes overlapped (the longest overlap consisted of 43 nucleotides between *ATP6* and *ATP8*). ATG and TAA were the most common initiation and termination codons, respectively. Incomplete termination codons were observed in *COX3, ND4,* and *Cyt-b* genes for all species (Tables S3–S6). The heavy strand contains all PCGs except ND6, which is on the light strand. GC% values were calculated as 36.9% for *M. tristrami*, 38.1% for *M. persicus*, 38% for *M. vinogradovi,* and 37.8% for *M. crassus.* The haplotype diversity (Hd) value was 1.000 in total (with 15 haplotypes in 15 samples), and the nucleotide diversity (Pi) value was 0.148, while the number of mutations and polymorphic sites were 8,767 and 6,408, respectively. Of the 6,408 polymorphic sites, 1,802 were singleton sites, and 4,606 were parsimony informative sites.

### Phylogenetic analyzes

**Figure 3:**
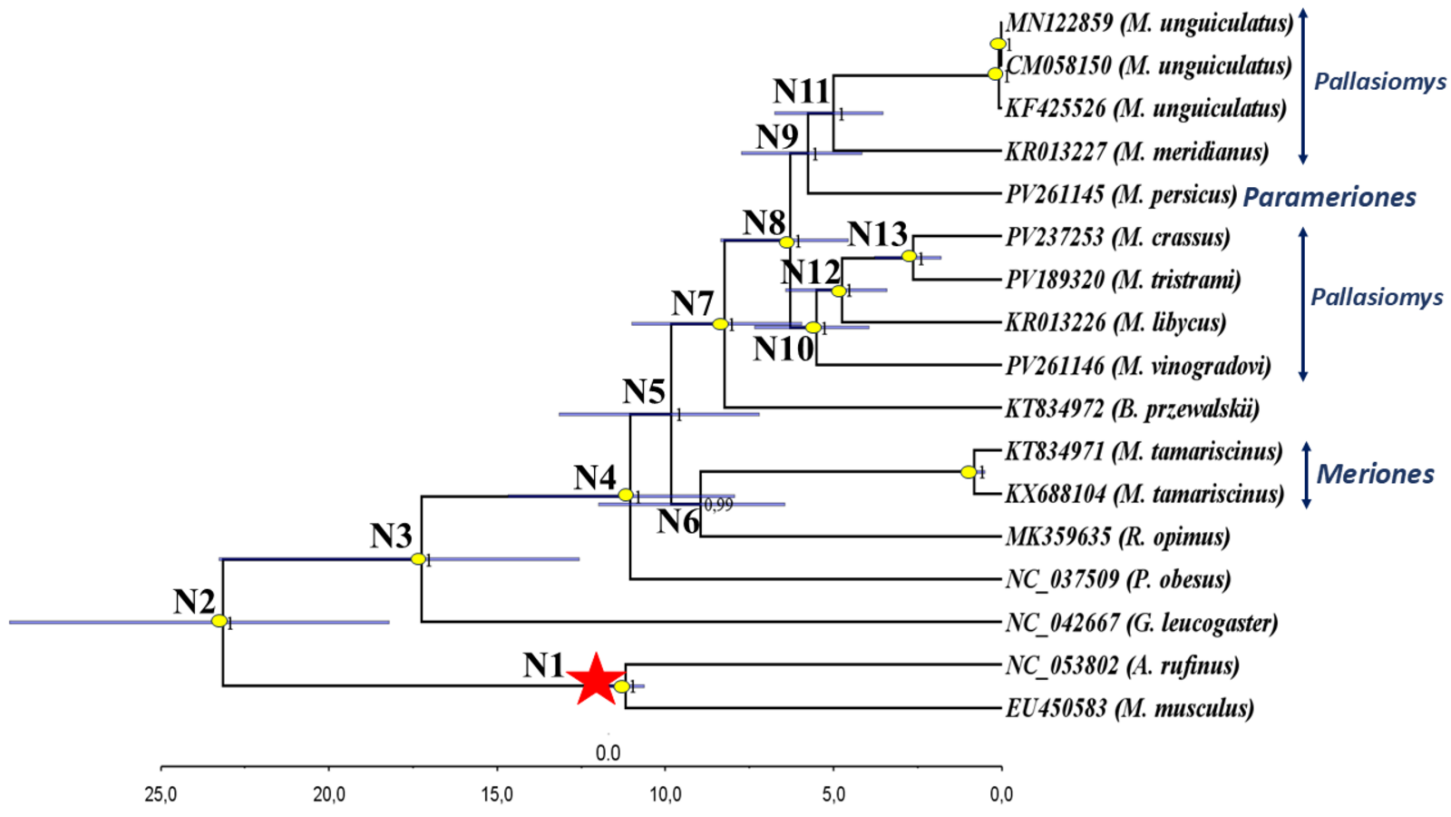
Bayesian and ML trees of *Meriones* species belonging to mitogenome data. Numbers at nodes represent posterior probabilities, and yellow circles indicate nodes with maximum likelihood bootstrap support ≥ 95%. Blue bars correspond to 95% highest posteri. Blue bars correspond to 95% highest posterior density (HPD) intervals for divergence time estimates. Divergence times at selected nodes were given in Table S7. The red star indicates the calibration node used to constrain divergence time (normal prior; mean = 11.2 Ma, SD = 0.3).

In complete mitogenome analyzes, mean genetic distance values based on the *p*-distance parameter were calculated between 7.04–16.59%. The lowest *d* value was between *M. tristrami-M. crassus* and the highest *d* value was between *M. meridianus-M. tamariscinus* (Table 1). In the BI tree (Fig. 3), after the split of *G. leucogaster* and *P. obesus* (pp (posterior probability) values are 1), *M. tamariscinus* gathered with *R. opimus* as the furthest lineage (pp: 0.99). Following diverging of *B. przewalskii* (pp: 1.00), two main lineages were emerged (pp: 1.00); the first lineage consisted of *M. meridianus, M. unguiculatus* and *M. persicus* (pp: 1.00) and the second lineage comprised of *M. tristrami, M. crassus, M. libycus* and *M. vinogradovi* (pp: 1.00). In the latter lineage, it could be observed that *M. tristrami* and *M. crassus* were closer than to remaining species (pp: 1.00). It could be also inferred that subgenus *Parameriones (M. persicus)* and subgenus *Pallasiomys* (other *Meriones* species) were not separated. In this respect, *Pallasiomys* exhibited a paraphyletic topology in the BI tree. Likewise, the position of *B. przewalskii* indicates the paraphyly of genus *Meriones* (Fig. 3). The divergence times of all studied species were dated back to 17.25 MYA (95% HPD: 12.56–23.27), as shown in Fig. 3 and Table S7. The ML tree exhibited the same topology with the bootstrap values between 58–100% (Fig. 3).

**Table 1:** Mean genetic distance (*d*) values (below the diagonal) of mitogenome data with the standard errors (above the diagonal).

	TRI	CRA	PER	VIN	MER	LIB	UNG	TAM	GER	BP	PO	RO
TRI		0.002	0.002	0.002	0.002	0.002	0.002	0.002	0.003	0.002	0.003	0.002
CRA	7.04			0.002	0.002	0.002	0.002	0.002	0.003	0.002	0.002	0.002
PER	12.72	12.32		0.002	0.002	0.002	0.002	0.002	0.003	0.002	0.002	0.003
VIN	11.35	11.06	12.64		0.002	0.002	0.002	0.002	0.003	0.002	0.003	0.002
MER	13.14	13.06	13.58	13.41		0.002	0.002	0.002	0.003	0.002	0.002	0.003
LIB	10.97	10.63	12.86	11.34	13.79		0.002	0.002	0.003	0.002	0.002	0.003
UNG	12.86	13.03	13.68	13.35	13.75	13.82		0.002	0.003	0.002	0.003	0.002
TAM	15.39	15.46	15.98	15.46	16.59	15.64	16.53		0.003	0.003	0.002	0.003
GER	20.18	20.18	20.69	20.52	21.15	20.60	20.92	20.32		0.003	0.003	0.003
BP	14.50	14.20	15.40	14.71	15.94	14.63	16.19	16.17	20.77		0.003	0.003
PO	15.59	15.36	16.21	15.50	16.67	15.71	16.65	15.92	20.05	16.05		0.003
RO	16.42	16.43	17.19	16.34	17.22	16.67	16.94	16.94	21.05	17.20	16.92	

**Notes.**

TRI *M. tristrami* CRA *M. crassus* PER *M. persicus* VIN *M. vinogradovi* LIB *M. libycus* MER *M. meridianus* UNG *M. unguiculatus* TAM *M. tamariscinus* GER *G. leucogaster* BP *B. przewalskii* PO *P. obesus* RO *R. opimus*

**Figure 4:**
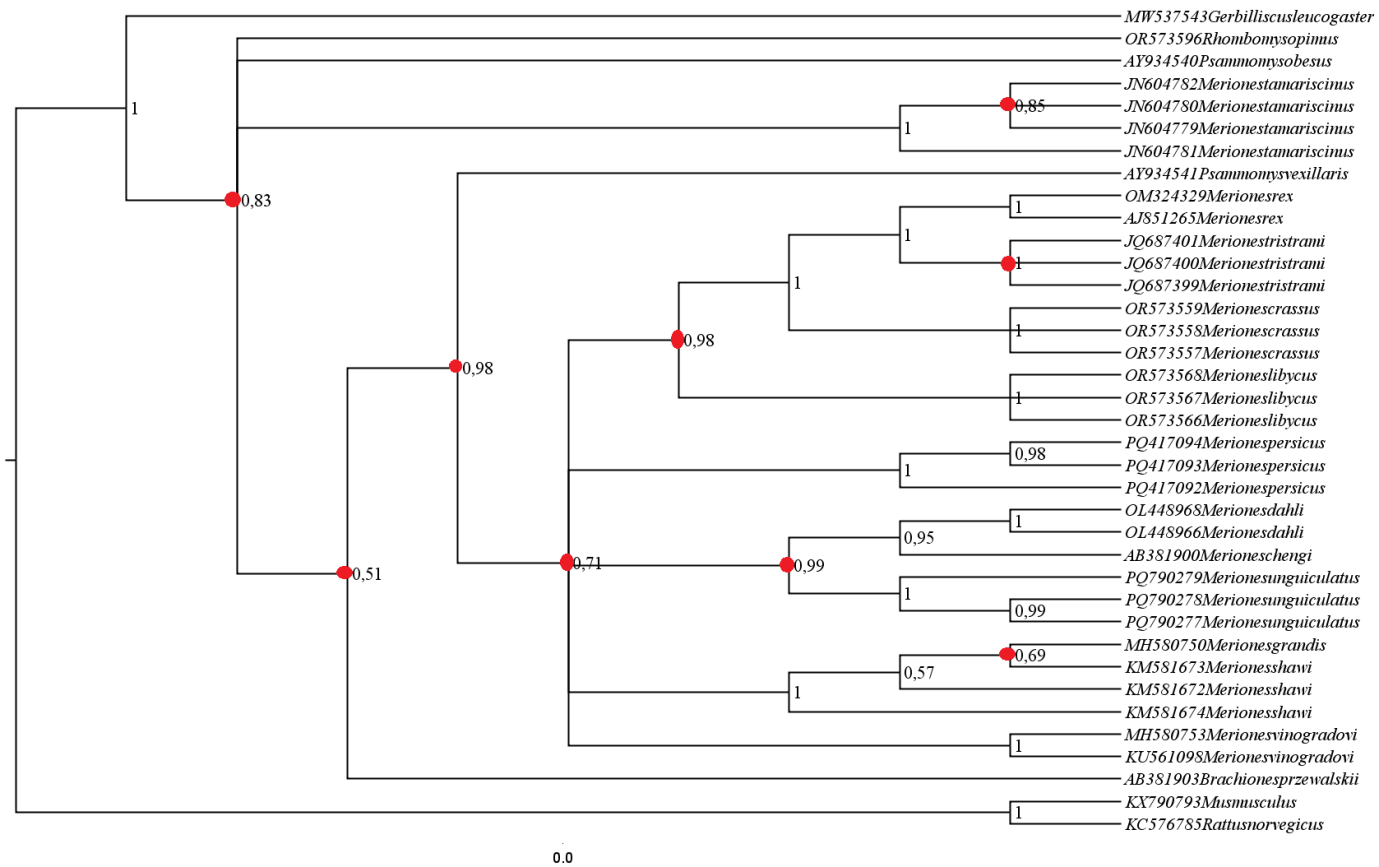
Bayesian and ML trees of *Meriones* species belonging to the *Cyt-b* data. Posterior probability (pp) values are shown with the numbers on nodes, and divergence times are indicated by the numbers on branches. Bootstrap values below 95% are indicated with red circles.

*Cyt-b* analyses yielded different results from the mitogenome in terms of *M. vinogradovi*’s position, with pp values ranging from 0.53 to 1.00 in the main branches. *M. vinogradovi* was located far from the *M. tristrami, M. crassus,* and *M. libycus* group. *B. pyrzewalskii* was the furthest species, and *P. vexillaris* was closer to *Meriones* species than *P. obesus* (Fig. 4). Also, *d* values ranged from 0.8% (*M. shawi-M. grandis*) to 18.4% (*M. tristrami-M. unguiculatus*) (Table 2).

## Discussion

### Comparison of the mitogenomes of Gerbillinae species

In the presented study, 16,412, 16,368, 16,452, and 16,485 base-pair regions were acquired from one sample each of *M. tristrami, M. persicus, M. vinogradovi,* and *M. crassus* for which complete mitogenome was given for the first time. Considering the data in Tables S3–S6, it could be seen that the differences in the lengths of some regions, such as *12S rRNA, 16S rRNA, tRNA-Asn, ATP synthase F0 subunit 8 (ATP8), tRNA-Arg, NADH dehydrogenase subunit 6 (ND6), tRNA-Pro,* and *Cyt-b,* in terms of one to six nucleotides. These small length differences may cumulatively contribute to the overall variation in mitogenome size between species. In addition, gene overlaps also play a role in the genome organization and total length. Overlapping genes can affect the total length because shared nucleotide sequences between adjacent genes reduce overall genome length compared to strictly non-overlapping arrangements. In the analyzed sequences, some genes overlapped, with the longest overlap (43 nucleotides) being between *ATP6* and *ATP8*, similar to other studies conducted by Ding et al. (2022) for *M. tamariscinus* and Li et al. (2016b) for *Meriones unguiculatus*. Length of protein-coding sequences (PCGs) was calculated as 11,408 bp—69.51% of the complete genome (*M. tristrami*), 11,417 bp—69.76% (*M. persicus*), 11,404 bp—69.31% (*M. vinogradovi*), and 11,408 bp—69.20% (*M. crassus*). These values are similar to *M. meridianus* (11.341 bp—69.25%, Luo & Liao (2016a)) and *M. libycus* (11.364 bp—69.54%, Luo & Liao (2016b)). GC content (36.9–38.1%) was also close and lower than AT content. In this case, it could be said that AT content is richer, in line with other studies on gerbils (*P. roborovskii*: 38.6% (Ding, Li & Liao, 2016); *M. meridianus*: 38.4% (Luo & Liao, 2016a), *M. libycus*: 38.4% (Luo & Liao, 2016b); *M. tamariscinus*: 36.8% (Li et al., 2016a); *M. unguiculatus*: 37% (Li et al., 2016b); *P. obesus*: 36.68% (Lan, Liu & Cao, 2018). Since two hydrogen bonds bind the AT pair while three bonds bind the GC pair, it was theorized that high AT content decreases the stability of the double helix structure of DNA (Yakovchuk, Protozanova & Frank-Kamenetskii, 2006), and this situation causes a higher mutation rate in rodents (Triant & De Woody, 2006). In addition, as compatible with typical mammals, the *12S rRNA* and the *16S rRNA* genes were located between the *tRNAPhe* and *tRNALeu* genes and were separated by the *tRNAVal* gene (Wolstenholme, 1992; Ding, Li & Liao, 2016).

**Table 2:** Mean genetic distance (*d*) values (below the diagonal) of *Cyt-b* data with the standard errors (above the diagonal).

	TRI	CRA	PER	VIN	MER	LIB	CHE	GRA	UNG	SHA	REX	TAM	GER	BP	PO	PV	RO
TRI		0.010	0.012	0.011	0.013	0.012	0.012	0.012	0.012	0.012	0.009	0.013	0.014	0.013	0.013	0.012	0.013
CRA	12.49		0.012	0.012	0.013	0.012	0.013	0.011	0.013	0.011	0.009	0.013	0.014	0.013	0.013	0.012	0.013
PER	17.72	12.85		0.012	0.012	0.012	0.012	0.012	0.013	0.012	0.013	0.013	0.015	0.013	0.013	0.013	0.014
VIN	15.60	11.58	12.90		0.012	0.012	0.012	0.012	0.014	0.012	0.012	0.013	0.015	0.013	0.013	0.012	0.013
MER	17.98	13.45	12.95	12.22		0.014	0.011	0.013	0.013	0.012	0.014	0.014	0.015	0.013	0.014	0.013	0.013
LIB	15.46	10.35	13.53	11.58	14.95		0.013	0.013	0.013	0.013	0.012	0.013	0.014	0.013	0.013	0.013	0.014
CHE	17.42	13.08	13.26	11.58	9.08	13.76		0.012	0.012	0.012	0.013	0.014	0.014	0.013	0.013	0.013	0.014
GRA	16.14	10.35	13.90	12.67	13.58	14.31	13.62		0.012	0.003	0.012	0.014	0.015	0.013	0.014	0.012	0.013
UNG	18.36	14.17	15.88	15.76	14.45	15.26	12.99	13.17		0.012	0.013	0.014	0.015	0.013	0.014	0.013	0.014
SHA	15.63	9.72	13.17	12.08	12.86	13.71	12.81	0.86	13.03		0.012	0.013	0.015	0.013	0.014	0.011	0.013
REX	11.58	5.18	13.76	11.04	15.09	11.38	13.76	11.72	15.92	11.35		0.013	0.015	0.014	0.014	0.013	0.013
TAM	18.23	14.88	15.34	14.48	16.35	14.07	15.43	16.11	17.00	15.34	14.41		0.015	0.014	0.014	0.013	0.014
GER	21.48	19.07	20.30	20.16	20.96	19.48	19.62	20.30	21.62	19.85	20.30	19.24		0.016	0.015	0.015	0.014
BP	19.24	15.40	16.62	14.99	15.90	15.80	15.12	15.40	15.58	15.21	17.57	15.16	20.98		0.013	0.013	0.013
PO	19.56	14.44	15.03	15.12	16.18	14.71	15.12	15.67	16.98	15.08	15.67	15.84	20.16	15.67		0.013	0.013
PV	17.92	13.08	14.44	12.94	14.27	13.76	14.85	11.99	14.71	11.26	14.99	14.88	20.30	15.12	12.81		0.013
RO	19.15	15.67	16.80	14.58	14.95	18.53	16.35	15.40	15.89	15.26	16.01	15.57	19.35	15.12	14.31	15.80	

**Notes.**

TRI *M. tristrami* CRA *M. crassus* PER *M. persicus* VIN *M. vinogradovi* LIB *M. libycus* CHE *M. chengi* GRA *M. grandis* MER *M. meridianus* UNG *M. unguiculatus* SHA *M. shawi* REX *M. rex* TAM *M. tamariscinus* GER *G. leucogaster* BP *B. przewalskii* PO *P. obesus* PV *P. vexillaris* RO *R. opimus*

### Phylogenetic analyzes of the genus *Meriones*

Regarding the studies on *Meriones* species recorded from Türkiye, it is seen that they mainly include morphological and allozyme data studies whose results contradict each other (Benazzou et al., 1984; Kefelioğlu, 1997; Yiğit, Kıvanç & Çolak, 1997; Yiğit, Kıvanç & Çolak, 1998; Yiğit et al., 2006b; Yiğit et al., 2013; Yiğit et al., 2018; Yiğit, Kıvanç & Çolak, 1998; Yiğit & Çolak, 1999; Darvish, 2009; Baydemir et al., 2011; Yazdi, Adriaens & Darvish, 2014), or molecular studies at species level (Dianat et al., 2017; Dianat et al., 2020; Yiğit et al., 2020). Molecular genetic studies evaluating inter-specific relationships are incongruent, although they included similar gene (*Cyt-b*) regions (Chevret & Dobigny, 2005; Ito et al., 2010; Nanova et al., 2020; Afzali & López-Antoñanzas, 2023).

The genus *Meriones* has a wide distribution area in arid and desert regions of the Middle East, Northern Africa, and Central Asia (Wilson & Reeder, 2005). The origin of the genus *Meriones* was calculated as 7.08 (Late Miocene) (Afzali & López-Antoñanzas, 2023) and 3.67 MYA (Early Pliocene) (Chevret & Dobigny, 2005) based on the *Cyt-b* region. In a way that coincides with Afzali & López-Antoñanzas (2023), the split time of the studied *Meriones* species was determined to be 11.04 MYA (95% HPD: 7.94–14.68) (Late Miocene) in this study. It is known that Quaternary climatic fluctuations in the Middle East and surrounding territories significantly impacted the speciation events (Hewitt, 2000); therefore, a rapid differentiation followed by the emergence of new gerbil species can be expected. However, gerbils are morphologically similar to each other, and thus, species identification is challenging (Yiğit, Kıvanç & Çolak, 1997; Yiğit, Kıvanç & Çolak, 1998; Yiğit et al., 2006a; Yiğit et al., 2018; Yiğit & Çolak, 1998; Yiğit & Çolak, 1999). Therefore, studies based on utilizing genetic markers are essential for elucidating the systematics and taxonomy of the genus *Meriones*. Based on *Cyt-b* and *12S rRNA* analyzes, *M. crassus* and *M. libycus* and *M. meridianus,* and *M. unguiculatus* are closely related gerbil species (Chevret & Dobigny, 2005). Ito et al. (2010) supported this finding using *Cyt-b* and *COII* markers. They suggested that *M. tamariscinus* is more related to *Brachiones, Rhombomys,* and *Psammomys* than the *Meriones* members. According to Nanova et al. (2020), Kimura-2 (Kimura, 1980) distances of the *Cyt-b* region between *Meriones* species (*M. meridianus, M. psammophilus*, and *M. penicilliger*) were found to be 8.1–11.8% (these values were 3.2–9.8%; (Nanova et al., 2024), whereas 8.9% (*M. libycus* and *M. arimalius*) and 5.4% (*M. rex* and *M. crassus*) distance values were also calculated. Alhajeri, Hunt & Steppan (2015) determined *M. libycus/M. tristrami/M. crassus/M. rex clade* based on the mitochondrial and nuclear data. Besides, *M. persicus* and *M. tamariscinus* were connected to the studied *Meriones* species; *Psammomys obesus, P. vexillaris, Brachiones przewalskii,* and *Rhombomys opimus* diverged from *Meriones*. Afzali & López-Antoñanzas (2023) determined two main lineages as strongly supported *M. libycus/M. tristrami/M. crassus* lineage and less supported *M. persicus/M. meridianus/M. vinogradovi/R. opimus*. Besides, Bouarakia et al. (2021) showed the close relationships between *M. tristrami* and *M. crassus, M. libycus, M. vinogradovi*, and *M. meridianus* and *M. unguiculatus* in *Cyt-b* analyzes. In studies examining the complete mitochondrial genome, Li et al. (2016a) defined that *M. unguiculatus* and *M. meridianus* were closely related, and *M. libycus* and *M. tamariscinus* were connected to this clade, respectively. In the study conducted by Luo & Liao (2016a), *M. meridianus* and *M. unguiculatus* were clustered in a single branch. Ding et al. (2022) suggested that *M. tamariscinus* was phylogenetically distant and had a close relationship with *Rhombomys opimus*.

According to the mitogenome results of this study, *M. tristrami* and *M. crassus* were found to be closely related species, consistent with Yiğit et al. (2013) and Afzali & López-Antoñanzas (2023); *M. libycus* and *M. vinogradovi* were connected to these two species. Similar to Chevret & Dobigny (2005), Ito et al. (2010), and Afzali & López-Antoñanzas (2023); while *M. meridianus, M. persicus,* and *M. unguiculatus* constituted a separate clade, *M. tamariscinus* was the most distant species, clustered with *R. opimus*. Furthermore, *B. przewalskii* is closer to *M. tristrami, M. crassus, M. libycus,* and *M. vinogradovi* lineage; besides, both *R. opimus* and *B. przewalskii* species were grouped with *Meriones* species. The fact that *Parameriones* and *Pallasiomys* subgenera of *Meriones* were not separated from each other confirms the view that *Meriones* is not monophyletic as suggested by different authors, and establishes paraphyletic trees in the phylogenetic approaches (Chevret & Dobigny, 2005; Ito et al., 2010; Yiğit et al., 2013; Alhajeri, Hunt & Steppan, 2015; Afzali & López-Antoñanzas, 2023). The presented study is the first mitochondrial DNA report that supports the paraphyly of the subgenus *Pallasiomys* concerning the placement of *M. persicus*, contradicting the previous studies. Specifically, the phylogenetic analyzes revealed that species assigned to *Pallasiomys,* such as *M. persicus,* did not form a separate, exclusive clade but instead clustered within a broader, paraphyletic topology of the genus *Meriones*. This indicates the uncertainty of the validity of these subgenera and suggests that the classification of *Meriones* needs re-evaluation.

The inconsistencies in results from previous studies (and this study’s *Cyt-b* results), despite mostly analyzing the same gene region (*Cyt-b*), arise primarily because relying on a single gene region provides limited genetic information and can lead to ambiguous or conflicting phylogenetic signals. The single gene, such as the *Cyt-b* region alone, may not capture the full evolutionary history or adequately resolve relationships among closely related species, especially in groups with rapid speciation or complex evolutionary patterns. This limitation highlights the necessity for new methods that utilize more comprehensive genetic data. Complete mitogenome analyzes not only give more information about gene regions but also present larger mitochondrial datasets (Boore, 2006; Dowton, Castro & Austin, 2002; Masta, McCall & Longhorn, 2010). In the light of the results presented, many well-supported branches with high reliability (pp values, mostly 1.00) were generated in the BI and ML trees (Fig. 3) to reveal the phylogeny of the gerbil species. Therefore, this study demonstrates the importance of complete mitogenome analyses in elucidating the systematics of controversial groups, especially the genus *Meriones*. On the other hand, due to some limitations, mitochondrial DNA analysis alone is insufficient for species identification; examples of these limitations include gene mixing, hybridization, incomplete lineage sorting, maternal inheritance, recombination, inconsistent mutation rate, and heteroplasmy (Rubinoff, Cameron & Will, 2006; Xing, Lin & Wu, 2025). It should be also mentioned that as molecular clock analyses based on a single calibration point in the outgroup may produce less reliable divergence time estimates (Benton & Donoghue, 2007; Ho & Phillips, 2009; Parham et al., 2012). Considering the low sample size of this study, further studies must be conducted with a larger sample size along with nuclear gene data.

## Conclusions

This study aimed to elucidate the phylogenetic relationships of gerbil species belonging to the genus *Meriones*, which have been a subject of debate for years. Within this scope, mitogenome analyzes were performed, as well as complete mitochondrial genome data of four *Meriones* species from Türkiye (*Meriones crassus, Meriones persicus, Meriones tristrami,* and *Meriones vinogradovi*) were characterized. Mitogenomes of these species were observed to be AT-rich, with the 36.9–38.1 GC% ratio. The BI and ML trees gave high-reliability results; *R. opimus* and *G. leucogaster* were the most distant species, *M. tamariscinus* was a sister taxon with *P. obesus*. *B. przewalskii* was connected to other *Meriones* species, proving the paraphyly of the genus *Meriones*. *M. meridianus, M. unguiculatus,* and *M. persicus* formed a distinct clade. *M. tristrami* and *M. crassus* were closely located, followed by *M. libycus* and *M. vinogradovi*. *M. persicus*’ position validates that subgenus *Pallasiomys* is paraphyletic. Also, 7.04–16.59% of mean genetic distance values were defined between *Meriones* species, and all the studied taxa started to diverge in the Late Miocene (11 MYA). It can also be concluded that complete mitochondrial genome studies can be more effective than single-gene analyses due to the larger and more informative dataset; however, this approach is not without limitations, including maternal inheritance and lack of recombination (Avise, 2000), representation of a single genetic locus with potential discordance due to introgression and incomplete lineage sorting (Ballard & Whitlock, 2004), and possible biases related to selective pressures and the presence of nuclear mitochondrial pseudogenes (NUMTs) (Bensasson et al., 2001).

## Supplemental Information

10.7717/peerj.21330/supp-1 Supplemental Information 1 GenBank Accession Numbers of the sequences utilized in this study

10.7717/peerj.21330/supp-2 Supplemental Information 2 NCBI-BLAST results of morphologically identified specimens based on Cyt-*b* marker

10.7717/peerj.21330/supp-3 Supplemental Information 3 Organization of the gene regions in the mitogenome of *M. tristrami*

10.7717/peerj.21330/supp-4 Supplemental Information 4 Organization of the gene regions in the mitogenome of *M. persicus*

10.7717/peerj.21330/supp-5 Supplemental Information 5 Organization of the gene regions in the mitogenome of *M. vinogradovi*

10.7717/peerj.21330/supp-6 Supplemental Information 6 Organization of the gene regions in the mitogenome of *M. crassus*

10.7717/peerj.21330/supp-7 Supplemental Information 7 Mitogenome characteristics of *M. persicus*

10.7717/peerj.21330/supp-8 Supplemental Information 8 Mitogenome characteristics of *M. vinogradovi*

10.7717/peerj.21330/supp-9 Supplemental Information 9 Mitogenome characteristics of *M. crassus*

10.7717/peerj.21330/supp-10 Supplemental Information 10 Genbank feature table of PV189320

10.7717/peerj.21330/supp-11 Supplemental Information 11 Genbank feature table of PV237253

10.7717/peerj.21330/supp-12 Supplemental Information 12 Genbank feature table of PV261145

10.7717/peerj.21330/supp-13 Supplemental Information 13 Genbank feature table of PV261146

10.7717/peerj.21330/supp-14 Supplemental Information 14 Raw sequence data of PV189320

10.7717/peerj.21330/supp-15 Supplemental Information 15 Raw sequence data of PV237253

10.7717/peerj.21330/supp-16 Supplemental Information 16 Raw sequence data of PV261145

10.7717/peerj.21330/supp-17 Supplemental Information 17 Raw sequence data of PV261146

10.7717/peerj.21330/supp-18 Supplemental Information 18 Divergence time estimates and corresponding 95% highest posterior density (HPD) intervals for all nodes inferred from Bayesian phylogenetic analysis

## Additional Information and Declarations

### Data Availability

The following information was supplied regarding data availability: The sequences are available in the Supplementary Files and at NCBI: PV237253.1, PV261145.1, PV189320.1, PV261146.1.
