# Supplementary material for: Complete mitochondrial genome analyzes of four gerbil species (Rodentia: Gerbillinae) distributed in Türkiye
Source: PeerJ. 2026 Jun 16;14:e21330. doi: 10.7717/peerj.21330 (PMC13281748; doi:10.7717/peerj.21330)
Supplement: Supplemental Information 1 [file peerj-14-21330-s001.docx]

Table S1 GenBank Accession Numbers of the sequences utilized in this study.

| **Species** | **GenBank Accession Number** | **Reference** |
| --- | --- | --- |
| **Mitogenome** |  |  |
| *M. crassus* | PV237253.1 | This study |
| *M. persicus* | PV261145.1 | This study |
| *M. tristrami* | PV189320.1 | This study |
| *M. vinogradovi* | PV261146.1 | This study |
| *M. tamariscinus* | KX688104.1,  KT834971.1 | Li et al., (2016a) |
| *M. meridianus* (*M. dahli*) | KR013227.1 | Luo & Liao (2016a) |
| *M. libycus* | KR013226.1 | Luo & Liao (2016b) |
| *M. unguiculatus* | KF425526.1  CM058150.1  MN122859.1 | Li et al., (2016b)  Brekke et al., (2023)  Mangaryan (Direct Submission) |
| *Rhombomys opimus* | MK359635.1 | Ding et al., (2022) |
| *Psammomys obesus* | NC_037509.1 | Lan et al., (2018) |
| *Brachiones przewalskii* | KT834972.1 | Ding et al., (2022) |
| *Gerbilliscus leucogaster* | NC_042667.1 | McDonough et al., (2018) |
| *Arvicanthis rufinus* | NC_053802.1 | Mikula et al. (Direct Submission) |
| *Mus musculus* | KF937876.1 | Zheng et al., (2014) |
| ***Cyt-b* Gene** |  |  |
| *M. crassus* | OR573557.1-OR573559.1 | Afzali & López-Antoñanzas (2023) |
| *M. persicus* | PQ417092.1-PQ417094.1 | Mahmoudi, Mostafavi & Kry²tufek (2025) |
| *M. tristrami* | JQ687399.1-JQ687401.1 | Yiğit et al., (2020) |
| *M. vinogradovi* | KU561098.1  MH580753.1 | Dianat et al., (2017)  Dianat et al., (2020) |
| *M. tamariscinus* | JN604779.1-JN604782.1 | Zhou et al. (Direct Submission) |
| *M. meridianus* (*M. dahli*) | OL448966.1, OL448968.1 | Nanova et al. (Direct Submission) |
| *M. libycus* | OR573566.1-OR573568.1 | Afzali & López-Antoñanzas (2023) |
| *M. unguiculatus* | PQ790277.1-PQ790279.1 | Blekhman et al., (2025) |
| *M. rex* | AJ851265.1  OM324329.1 | Chevret & Dobigny, (2005)  Li et al., (2023) |
| *M. shawi* | KM581672.1-KM581674.1 | Lalis et al., (2016) |
| *M. chengi* | AB381900.1 | Afzali & López-Antoñanzas (2023) |
| *M. grandis* | MH580750.1 | Dianat et al., (2020) |
| *Rhombomys opimus* | OR573596.1 | Afzali & López-Antoñanzas (2023) |
| *Psammomys obesus* | AY934540.1 | Mostafa et al., (2006) |
| *Psammomys vexillaris* | AY934541.1 | Mostafa et al., (2006) |
| *Brachiones przewalskii* | AB381903.1 | Ito et al., (2010) |
| *Gerbilliscus leucogaster* | MW537543.1 | Krásová et al., (2021) |
| *Rattus norvegicus* | KC576785.1 | Yao et al. (Direct Submission) |
| *Mus musculus* | KX790793.1 | Bibi et al., (2017) |
