# Supplementary material for: Complete mitochondrial genome analyzes of four gerbil species (Rodentia: Gerbillinae) distributed in Türkiye
Source: PeerJ. 2026 Jun 16;14:e21330. doi: 10.7717/peerj.21330 (PMC13281748; doi:10.7717/peerj.21330)
Supplement: Supplemental Information 2 [file peerj-14-21330-s002.docx]

Table S2 NCBI-BLAST results of morphologically identified specimens based on Cyt-*b* marker

| **Species** | **Max Score** | **Total Score** | **Query Cover** | **E value** | **Percent Identity** | **Accession Length** | **Accession Number** |
| --- | --- | --- | --- | --- | --- | --- | --- |
| *M. tristrami* | 2032 | 2032 | 99% | 0.0 | 99.03% | 1133 | KR089032.1 |
| *M. crassus* | 1871 | 1871 | 100% | 0.0 | 96.32% | 1139 | KR089028.1 |
| *M. persicus* | 2065 | 2065 | 99% | 0.0 | 99.56% | 1133 | PQ417070.1 |
| *M. vinogradovi* | 1500 | 1500 | 77% | 0.0 | 97.49% | 878 | KU561098.1 |
