# Supplementary material for: Complete mitochondrial genome analyzes of four gerbil species (Rodentia: Gerbillinae) distributed in Türkiye
Source: PeerJ. 2026 Jun 16;14:e21330. doi: 10.7717/peerj.21330 (PMC13281748; doi:10.7717/peerj.21330)
Supplement: Supplemental Information 3 [file peerj-14-21330-s003.docx]

Table S3. Organization of the gene regions in the mitogenome of *M. tristrami*

| **Start-End** | **Length (bp)** | **Direction** | **Type** | **Gene_name** | **Gene_product** | **Total_freq_occurred** |
| --- | --- | --- | --- | --- | --- | --- |
| 1-68 | 68 | H | tRNA | trnF(gaa) | tRNA-Phe | 1 |
| 68-1019 | 952 | H | rRNA | s-rRNA | 12S ribosomal RNA | 1 |
| 1020-1086 | 67 | H | tRNA | trnV(uac) | tRNA-Val | 1 |
| 1085-2670 | 1586 | H | rRNA | l-rRNA | 16S ribosomal RNA | 1 |
| 2669-2743 | 75 | H | tRNA | trnL(uaa) | tRNA-Leu | 2 |
| 2744-3698 | 955 | H | CDS | ND1 | NADH dehydrogenase subunit 1 | 1 |
| 3699-3766 | 68 | H | tRNA | trnI(gau) | tRNA-Ile | 1 |
| 3764-3836 | 73 | L | tRNA | trnQ(uug) | tRNA-Gln | 1 |
| 3847-3915 | 69 | H | tRNA | trnM(cau) | tRNA-Met | 1 |
| 3919- 4955 | 1037 | H | CDS | ND2 | NADH dehydrogenase subunit 2 | 1 |
| 4956-5020 | 65 | H | tRNA | trnW(uca) | tRNA-Trp | 1 |
| 5023-5091 | 69 | L | tRNA | trnA(ugc) | tRNA-Ala | 1 |
| 5142-5212 | 71 | L | tRNA | trnN(guu) | tRNA-Asn | 1 |
| 5244-5310 | 67 | L | tRNA | trnC(gca) | tRNA-Cys | 1 |
| 5311-5378 | 68 | L | tRNA | trnY(gua) | tRNA-Tyr | 1 |
| 5379-6923 | 1545 | H | CDS | COX1 | cytochrome c oxidase subunit I | 1 |
| 6921-6989 | 69 | L | tRNA | trnS(uga) | tRNA-Ser | 2 |
| 6993-7061 | 69 | H | tRNA | trnD(guc) | tRNA-Asp | 1 |
| 7063-7749 | 687 | H | CDS | COX2 | cytochrome c oxidase subunit II | 1 |
| 7750-7814 | 65 | H | tRNA | trnK(uuu) | tRNA-Lys | 1 |
| 7817-8020 | 204 | H | CDS | ATP8 | ATP synthase F0 subunit 8 | 1 |
| 7978-8658 | 681 | H | CDS | ATP6 | ATP synthase F0 subunit 6 | 1 |
| 8658-9442 | 785 | H | CDS | COX3 | cytochrome c oxidase subunit III | 1 |
| 9442-9509 | 68 | H | tRNA | trnG(ucc) | tRNA-Gly | 1 |
| 9510-9857 | 348 | H | CDS | ND3 | NADH dehydrogenase subunit 3 | 1 |
| 9863-9929 | 67 | H | tRNA | trnR(ucg) | tRNA-Arg | 1 |
| 9931-10227 | 297 | H | CDS | ND4L | NADH dehydrogenase subunit 4L | 1 |
| 10221-11598 | 1378 | H | CDS | ND4 | NADH dehydrogenase subunit 4 | 1 |
| 11599-11667 | 69 | H | tRNA | trnH(gug) | tRNA-His | 1 |
| 11668-11727 | 60 | H | tRNA | trnS(gcu) | tRNA-Ser | 2 |
| 11727-11794 | 68 | H | tRNA | trnL(uag) | tRNA-Leu | 2 |
| 11795-13607 | 1813 | H | CDS | ND5 | NADH dehydrogenase subunit 5 | 1 |
| 13608-14126 | 519 | L | CDS | ND6 | NADH dehydrogenase subunit 6 | 1 |
| 14127-14195 | 69 | L | tRNA | trnE(uuc) | tRNA-Glu | 1 |
| 14200-15343 | 1144 | H | CDS | CYT-B | cytochrome b | 1 |
| 15344-15410 | 67 | H | tRNA | trnT(ugu) | tRNA-Thr | 1 |
| 15411-15479 | 69 | L | tRNA | trnP(ugg) | tRNA-Pro | 1 |
| 15480-16412 | 933 | H | NCCR | Control Region | - | 1 |
