# Supplementary material for: Complete mitochondrial genome analyzes of four gerbil species (Rodentia: Gerbillinae) distributed in Türkiye
Source: PeerJ. 2026 Jun 16;14:e21330. doi: 10.7717/peerj.21330 (PMC13281748; doi:10.7717/peerj.21330)
Supplement: Supplemental Information 4 [file peerj-14-21330-s004.docx]

Table S4. Organization of the gene regions in the mitogenome of *M. persicus*

| **Start-End** | **Length (bp)** | **Direction** | **Type** | **Gene_name** | **Gene_product** | **Total_freq_occurred** |
| --- | --- | --- | --- | --- | --- | --- |
| 1-67 | 67 | H | tRNA | trnF(gaa) | tRNA-Phe | 1 |
| 68-1018 | 951 | H | rRNA | s-rRNA | 12S ribosomal RNA | 1 |
| 1019-1085 | 67 | H | tRNA | trnV(uac) | tRNA-Val | 1 |
| 1084-2668 | 1585 | H | rRNA | l-rRNA | 16S ribosomal RNA | 1 |
| 2667-2741 | 75 | H | tRNA | trnL(uaa) | tRNA-Leu | 2 |
| 2742-3696 | 955 | H | CDS | ND1 | NADH dehydrogenase subunit 1 | 1 |
| 3697-3764 | 68 | H | tRNA | trnI(gau) | tRNA-Ile | 1 |
| 3762-3834 | 73 | L | tRNA | trnQ(uug) | tRNA-Gln | 1 |
| 3843-3911 | 69 | H | tRNA | trnM(cau) | tRNA-Met | 1 |
| 3914-4950 | 1037 | H | CDS | ND2 | NADH dehydrogenase subunit 2 | 1 |
| 4951-5015 | 65 | H | tRNA | trnW(uca) | tRNA-Trp | 1 |
| 5026-5094 | 69 | L | tRNA | trnA(ugc) | tRNA-Ala | 1 |
| 5101-5172 | 72 | L | tRNA | trnN(guu) | tRNA-Asn | 1 |
| 5206-5272 | 67 | L | tRNA | trnC(gca) | tRNA-Cys | 1 |
| 5273-5339 | 67 | L | tRNA | trnY(gua) | tRNA-Tyr | 1 |
| 5341-6885 | 1545 | H | CDS | COX1 | cytochrome c oxidase subunit I | 1 |
| 6883-6951 | 69 | L | tRNA | trnS(uga) | tRNA-Ser | 2 |
| 6955-7023 | 69 | H | tRNA | trnD(guc) | tRNA-Asp | 1 |
| 7025-7711 | 687 | H | CDS | COX2 | cytochrome c oxidase subunit II | 1 |
| 7712-7776 | 65 | H | tRNA | trnK(uuu) | tRNA-Lys | 1 |
| 7779-7985 | 207 | H | CDS | ATP8 | ATP synthase F0 subunit 8 | 1 |
| 7940-8620 | 681 | H | CDS | ATP6 | ATP synthase F0 subunit 6 | 1 |
| 8620-9404 | 785 | H | CDS | COX3 | cytochrome c oxidase subunit III | 1 |
| 9404-9471 | 68 | H | tRNA | trnG(ucc) | tRNA-Gly | 1 |
| 9472-9819 | 348 | H | CDS | ND3 | NADH dehydrogenase subunit 3 | 1 |
| 9828-9895 | 68 | H | tRNA | trnR(ucg) | tRNA-Arg | 1 |
| 9897-10193 | 298 | H | CDS | ND4L | NADH dehydrogenase subunit 4L | 1 |
| 10187-11564 | 1378 | H | CDS | ND4 | NADH dehydrogenase subunit 4 | 1 |
| 11565-11633 | 69 | H | tRNA | trnH(gug) | tRNA-His | 1 |
| 11634-11693 | 60 | H | tRNA | trnS(gcu) | tRNA-Ser | 2 |
| 11693-11760 | 68 | H | tRNA | trnL(uag) | tRNA-Leu | 2 |
| 11761-13572 | 1812 | H | CDS | ND5 | NADH dehydrogenase subunit 5 | 1 |
| 13574-14098 | 525 | L | CDS | ND6 | NADH dehydrogenase subunit 6 | 1 |
| 14099-14167 | 69 | L | tRNA | trnE(uuc) | tRNA-Glu | 1 |
| 14172-15315 | 1144 | H | CDS | CYT-B | cytochrome b | 1 |
| 15316-15382 | 68 | H | tRNA | trnT(ugu) | tRNA-Thr | 1 |
| 15383-15451 | 69 | L | tRNA | trnP(ugg) | tRNA-Pro | 1 |
| 15452-16368 | 917 | H | NCCR | Control Region | - | 1 |
