# Supplementary material for: Complete mitochondrial genome analyzes of four gerbil species (Rodentia: Gerbillinae) distributed in Türkiye
Source: PeerJ. 2026 Jun 16;14:e21330. doi: 10.7717/peerj.21330 (PMC13281748; doi:10.7717/peerj.21330)
Supplement: Supplemental Information 5 [file peerj-14-21330-s005.docx]

Table S5 Organization of the gene regions in the mitogenome of *M. vinogradovi*

| **Start-End** | **Length (bp)** | **Direction** | **Type** | **Gene_name** | **Gene_product** | | **Total_freq_occurred** |
| --- | --- | --- | --- | --- | --- | --- | --- |
| 1-67 | 67 | H | tRNA | trnF(gaa) | tRNA-Phe | 1 | |
| 68-1018 | 951 | H | rRNA | s-rRNA | 12S ribosomal RNA | 1 | |
| 1019-1085 | 67 | H | tRNA | trnV(uac) | tRNA-Val | 1 | |
| 1086-2661 | 1575 | H | rRNA | l-rRNA | 16S ribosomal RNA | 1 | |
| 2662-2736 | 75 | H | tRNA | trnL(uaa) | tRNA-Leu | 2 | |
| 2722-3691 | 970 | H | CDS | ND1 | NADH dehydrogenase subunit 1 | 1 | |
| 3692-3759 | 68 | H | tRNA | trnI(gau) | tRNA-Ile | 1 | |
| 3762-3834 | 73 | L | tRNA | trnQ(uug) | tRNA-Gln | 1 | |
| 3840-3909 | 70 | H | tRNA | trnM(cau) | tRNA-Met | 1 | |
| 3913-4949 | 1037 | H | CDS | ND2 | NADH dehydrogenase subunit 2 | 1 | |
| 4950-5014 | 65 | H | tRNA | trnW(uca) | tRNA-Trp | 1 | |
| 5017-5085 | 69 | L | tRNA | trnA(ugc) | tRNA-Ala | 1 | |
| 5091-5162 | 72 | L | tRNA | trnN(guu) | tRNA-Asn | 1 | |
| 5195-5261 | 67 | L | tRNA | trnC(gca) | tRNA-Cys | 1 | |
| 5262-5328 | 67 | L | tRNA | trnY(gua) | tRNA-Tyr | 1 | |
| 5330-6874 | 1545 | H | CDS | COX1 | cytochrome c oxidase subunit I | 1 | |
| 6872-6940 | 69 | L | tRNA | trnS(uga) | tRNA-Ser | 2 | |
| 6944-7012 | 69 | H | tRNA | trnD(guc) | tRNA-Asp | 1 | |
| 7014-7700 | 687 | H | CDS | COX2 | cytochrome c oxidase subunit II | 1 | |
| 7701-7765 | 65 | H | tRNA | trnK(uuu) | tRNA-Lys | 1 | |
| 7770-7971 | 202 | H | CDS | ATP8 | ATP synthase F0 subunit 8 | 1 | |
| 7929-8608 | 680 | H | CDS | ATP6 | ATP synthase F0 subunit 6 | 1 | |
| 8609-9392 | 784 | H | CDS | COX3 | cytochrome c oxidase subunit III | 1 | |
| 9393-9460 | 68 | H | tRNA | trnG(ucc) | tRNA-Gly | 1 | |
| 9461-9808 | 348 | H | CDS | ND3 | NADH dehydrogenase subunit 3 | 1 | |
| 9820-9886 | 67 | H | tRNA | trnR(ucg) | tRNA-Arg | 1 | |
| 9888-10184 | 297 | H | CDS | ND4L | NADH dehydrogenase subunit 4L | 1 | |
| 10178-11555 | 1378 | H | CDS | ND4 | NADH dehydrogenase subunit 4 | 1 | |
| 11556-11624 | 69 | H | tRNA | trnH(gug) | tRNA-His | 1 | |
| 11625-11684 | 60 | H | tRNA | trnS(gcu) | tRNA-Ser | 2 | |
| 11684-11751 | 68 | H | tRNA | trnL(uag) | tRNA-Leu | 2 | |
| 11752-13563 | 1812 | H | CDS | ND5 | NADH dehydrogenase subunit 5 | 1 | |
| 13565-14083 | 519 | L | CDS | ND6 | NADH dehydrogenase subunit 6 | 1 | |
| 14084-14152 | 69 | L | tRNA | trnE(uuc) | tRNA-Glu | 1 | |
| 14157-15296 | 1140 | H | CDS | CYT-B | cytochrome b | 1 | |
| 15301-15367 | 67 | H | tRNA | trnT(ugu) | tRNA-Thr | 1 | |
| 15368-15437 | 70 | L | tRNA | trnP(ugg) | tRNA-Pro | 1 | |
| 15438-16452 | 1015 | H | NCCR | Control Region | - | 1 | |
