# Supplementary material for: Complete mitochondrial genome analyzes of four gerbil species (Rodentia: Gerbillinae) distributed in Türkiye
Source: PeerJ. 2026 Jun 16;14:e21330. doi: 10.7717/peerj.21330 (PMC13281748; doi:10.7717/peerj.21330)
Supplement: Supplemental Information 6 [file peerj-14-21330-s006.docx]

Table S6. Organization of the gene regions in the mitogenome of *M. crassus*

| **Start-End** | **Length (bp)** | **Direction** | **Type** | **Gene_name** | **Gene_product** | **Total_freq_occurred** |
| --- | --- | --- | --- | --- | --- | --- |
| 504-573 | 70 | **+** | tRNA | trnP(ugg) | tRNA-Pro | 1 |
| 570-640 | 71 | - | tRNA | trnT(ugu) | tRNA-Thr | 1 |
| 640-1784 | 1145 | - | CDS | CYT-B | cytochrome b | 1 |
| 1788-1857 | 70 | + | tRNA | trnE(uuc) | tRNA-Glu | 1 |
| 1857-2376 | 520 | + | CDS | ND6 | NADH dehydrogenase subunit 6 | 1 |
| 2377-4189 | 1813 | - | CDS | ND5 | NADH dehydrogenase subunit 5 | 1 |
| 4189-4257 | 69 | - | tRNA | trnL(uag) | tRNA-Leu | 2 |
| 4256-4317 | 62 | - | tRNA | trnS(gcu) | tRNA-Ser | 2 |
| 4317-4386 | 70 | - | tRNA | trnH(gug) | tRNA-His | 1 |
| 4386-5764 | 1379 | - | CDS | ND4 | NADH dehydrogenase subunit 4 | 1 |
| 5757-6054 | 298 | - | CDS | ND4L | NADH dehydrogenase subunit 4L | 1 |
| 6055-6122 | 68 | - | tRNA | trnR(ucg) | tRNA-Arg | 1 |
| 6127-6475 | 349 | - | CDS | ND3 | NADH dehydrogenase subunit 3 | 1 |
| 6475-6543 | 69 | - | tRNA | trnG(ucc) | tRNA-Gly | 1 |
| 6542-7327 | 786 | - | CDS | COX3 | cytochrome c oxidase subunit III | 1 |
| 7326-8007 | 682 | - | CDS | ATP6 | ATP synthase F0 subunit 6 | 1 |
| 7964-8168 | 205 | - | CDS | ATP8 | ATP synthase F0 subunit 8 | 1 |
| 8170-8235 | 66 | - | tRNA | trnK(uuu) | tRNA-Lys | 1 |
| 8235-8922 | 688 | - | CDS | COX2 | cytochrome c oxidase subunit II | 1 |
| 8923-8992 | 70 | - | tRNA | trnD(guc) | tRNA-Asp | 1 |
| 8995-9064 | 70 | + | tRNA | trnS(uga) | tRNA-Ser | 2 |
| 9061-10606 | 1546 | - | CDS | COX1 | cytochrome c oxidase subunit I | 1 |
| 10607-10674 | 68 | + | tRNA | trnY(gua) | tRNA-Tyr | 1 |
| 10674-10741 | 68 | + | tRNA | trnC(gca) | tRNA-Cys | 1 |
| 10772-10843 | 72 | + | tRNA | trnN(guu) | tRNA-Asn | 1 |
| 10850-10919 | 70 | + | tRNA | trnA(ugc) | tRNA-Ala | 1 |
| 10921-10986 | 66 | - | tRNA | trnW(uca) | tRNA-Trp | 1 |
| 10985-12023 | 1039 | - | CDS | ND2 | NADH dehydrogenase subunit 2 | 1 |
| 12027-12096 | 70 | - | tRNA | trnM(cau) | tRNA-Met | 1 |
| 12107-12179 | 73 | + | tRNA | trnQ(uug) | tRNA-Gln | 1 |
| 12176-12244 | 69 | - | tRNA | trnI(gau) | tRNA-Ile | 1 |
| 12242-13199 | 958 | - | CDS | ND1 | NADH dehydrogenase subunit 1 | 1 |
| 13199-13274 | 76 | - | tRNA | trnL(uaa) | tRNA-Leu | 2 |
| 13272-14857 | 1586 | - | rRNA | l-rRNA | 16S ribosomal RNA | 1 |
| 14855-14922 | 68 | - | tRNA | trnV(uac) | tRNA-Val | 1 |
| 14922-15873 | 952 | - | rRNA | s-rRNA | 12S ribosomal RNA | 1 |
| 15873-15940 | 68 | - | tRNA | trnF(gaa) | tRNA-Phe | 1 |
| 15940-16485 | 546 | **-** | NCCR | Control Region | - | 1 |
