# Supplementary figures and images for: Complete mitochondrial genome analyzes of four gerbil species (Rodentia: Gerbillinae) distributed in Türkiye
Source: PeerJ. 2026 Jun 16;14:e21330. doi: 10.7717/peerj.21330 (PMC13281748; doi:10.7717/peerj.21330)

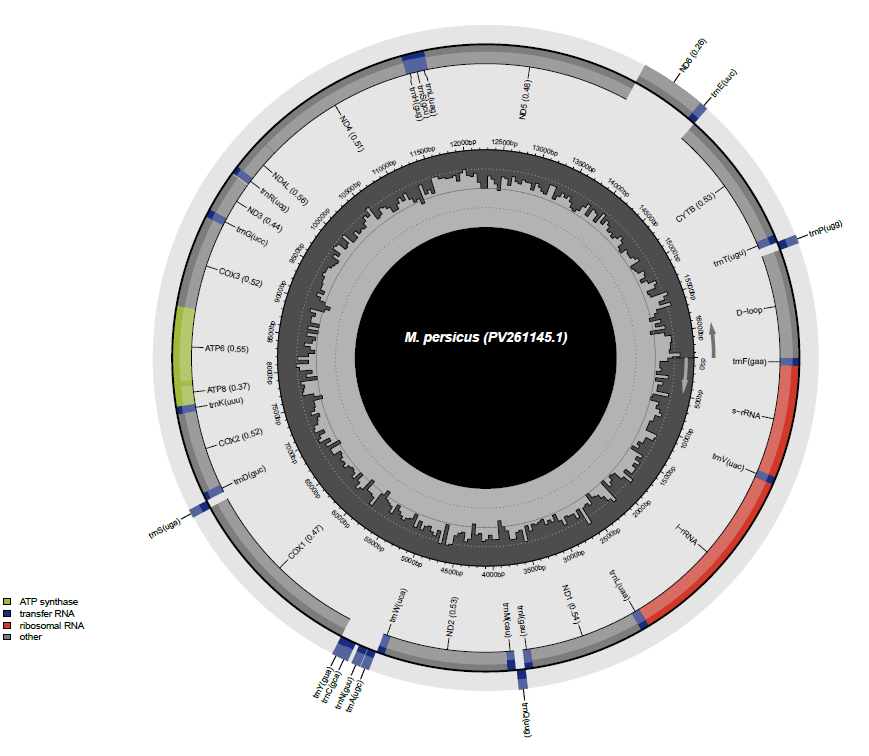

Supplement: Supplemental Information 7 [file peerj-14-21330-s007.png]

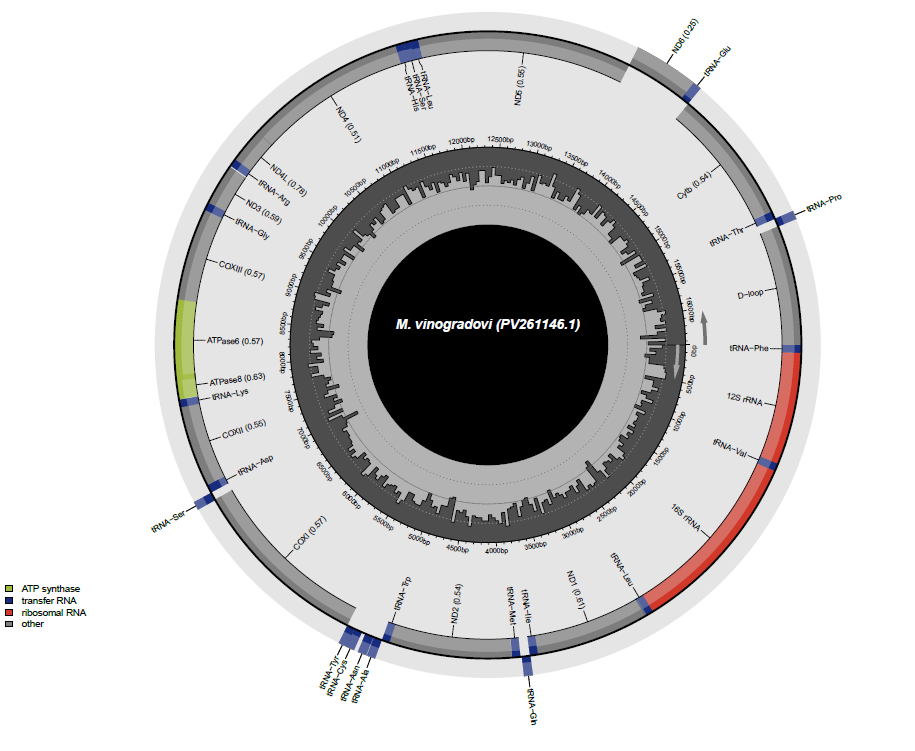

Supplement: Supplemental Information 8 [file peerj-14-21330-s008.png]

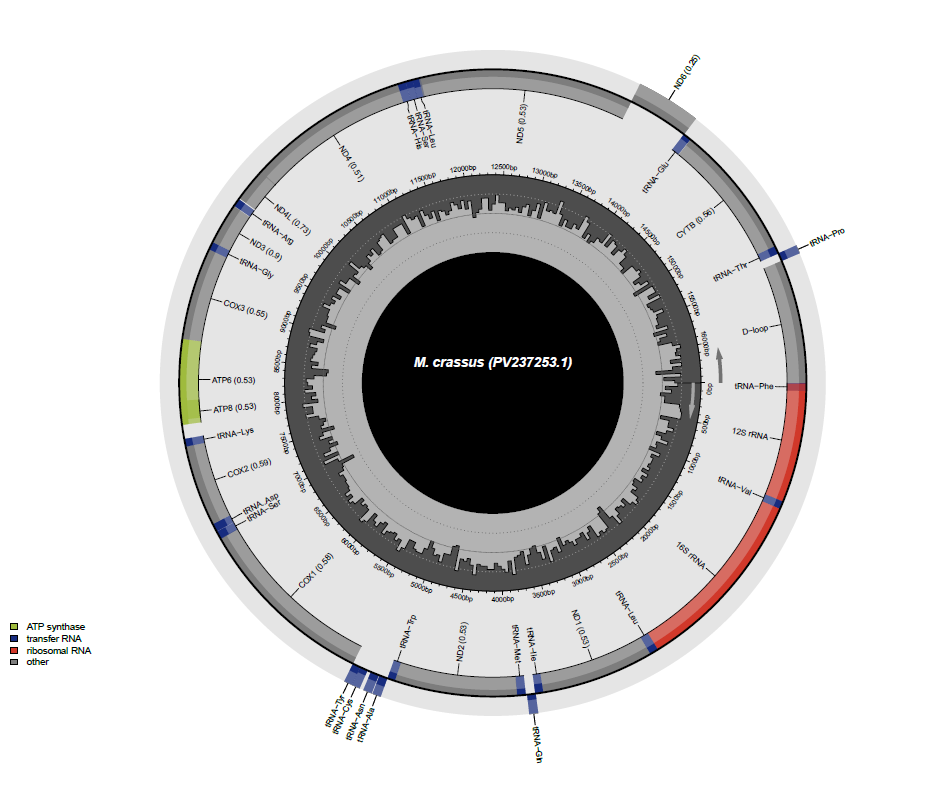

Supplement: Supplemental Information 9 [file peerj-14-21330-s009.png]
