# Supplementary material for: Complete mitochondrial genome analyzes of four gerbil species (Rodentia: Gerbillinae) distributed in Türkiye
Source: PeerJ. 2026 Jun 16;14:e21330. doi: 10.7717/peerj.21330 (PMC13281748; doi:10.7717/peerj.21330)
Supplement: Supplemental Information 18 [file peerj-14-21330-s018.docx]

Table S7. Divergence time estimates and corresponding 95% highest posterior density (HPD) intervals for all nodes inferred from Bayesian phylogenetic analysis.

| **Node ID** | **Taxon/Clade** | **Median age (MYA)** | **95% HPD (MYA)** |
| --- | --- | --- | --- |
| N1 | Calibration node (*Mus-Arvicanthis*) | 11.18 | 10.62–11.81 |
| N2 | Studied species-*Mus*/*Arvicanthis* split | 23.16 | 18.21–29.50 |
| N3 | *G. leucogaster* split | 17.25 | 12.56–23.27 |
| N4 | *P. obesus* split | 11.04 | 7.94–14.68 |
| N5 | *R. opimus/M. tamariscinus* -other taxa split | 9.83 | 7.21–13.15 |
| N6 | *R. opimus-M. tamariscinus* split | 8.96 | 6.44–11.98 |
| N7 | *B. przewalskii* split | 8.24 | 5.95–10.99 |
| N8 | *Parameriones-Pallasiomys* ingroup split | 6.28 | 4.56–8.35 |
| N9 | *M. persicus-M. unguiculatus/M. meridianus* split | 5.76 | 4.16–7.74 |
| N10 | *M. vinogradovi* split | 5.50 | 3.95–7.35 |
| N11 | *M. unguiculatus/M. meridianus* split | 4.99 | 3.53–6.75 |
| N12 | *M. libycus-* *M. crassus/M. tristrami* split | 4.75 | 3.41–6.42 |
| N13 | *M. crassus-M. tristrami* split | 2.63 | 1.79–3.77 |
|  |  |  |  |
|  |  |  |  |
|  |  |  |  |
|  |  |  |  |
